# Supplementary material for: Comprehensive Analysis of hsa-miR-654-5p’s Tumor-Suppressing Functions
Source: Int J Mol Sci. 2022 Jun 8;23(12):6411. doi: 10.3390/ijms23126411 (PMC9224266; doi:10.3390/ijms23126411)
Supplement: Supplementary file 1 [file ijms-23-06411-s001.zip › Supp. Info. 1 IJMS_R2.pdf]

Supplementary Materials

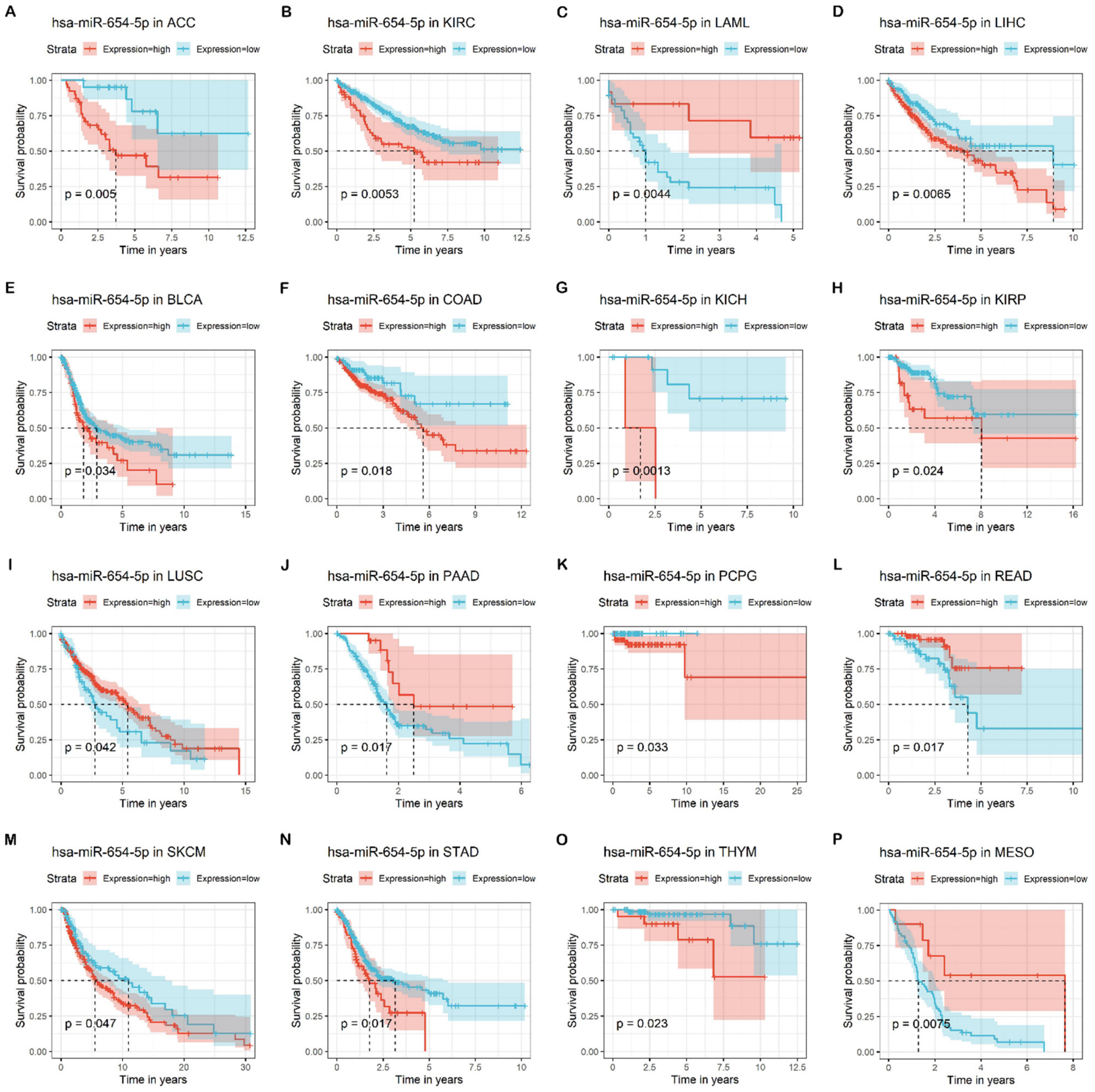

Figure S1. Survival analysis of hsa-miR-654-5p in the pan-cancer dataset.

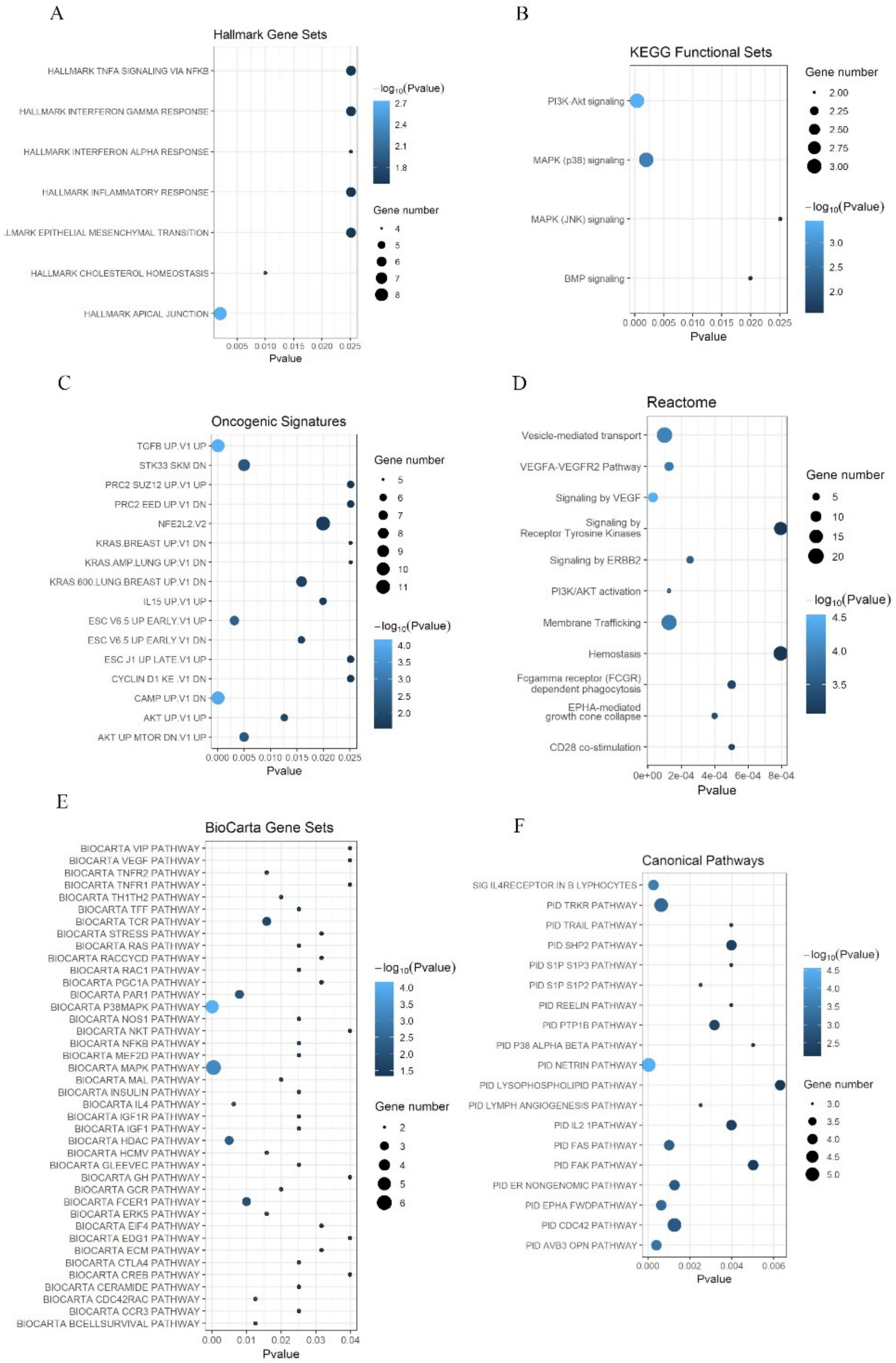

Figure S2. enrichment analysis of 275 overlapping predicted targets of miR-622. the 275 predicted genes of miR-654-5p were chosen to perform Hallmark, KEGG functional sets, Oncogenic signatures, Reactome, BioCarta and Canonical pathway enrichment analysis. Each bubble represents

a term, and its size reprensent the counts of involved genes. Lighter colors indicate smaller P values. (A) enriched items for hallmark gene sets (p<0.05). (B) enriched items for KEGG functional sets (p<0.05). (C) enriched items for Oncogenic signatures (p<0.05). (D) enriched items for Reactome (p<0.001). (E) enriched items for BioCarta Gene Sets (p<0.05). (F) enriched items for Canonical Pathways (p<0.01)[1, 2].

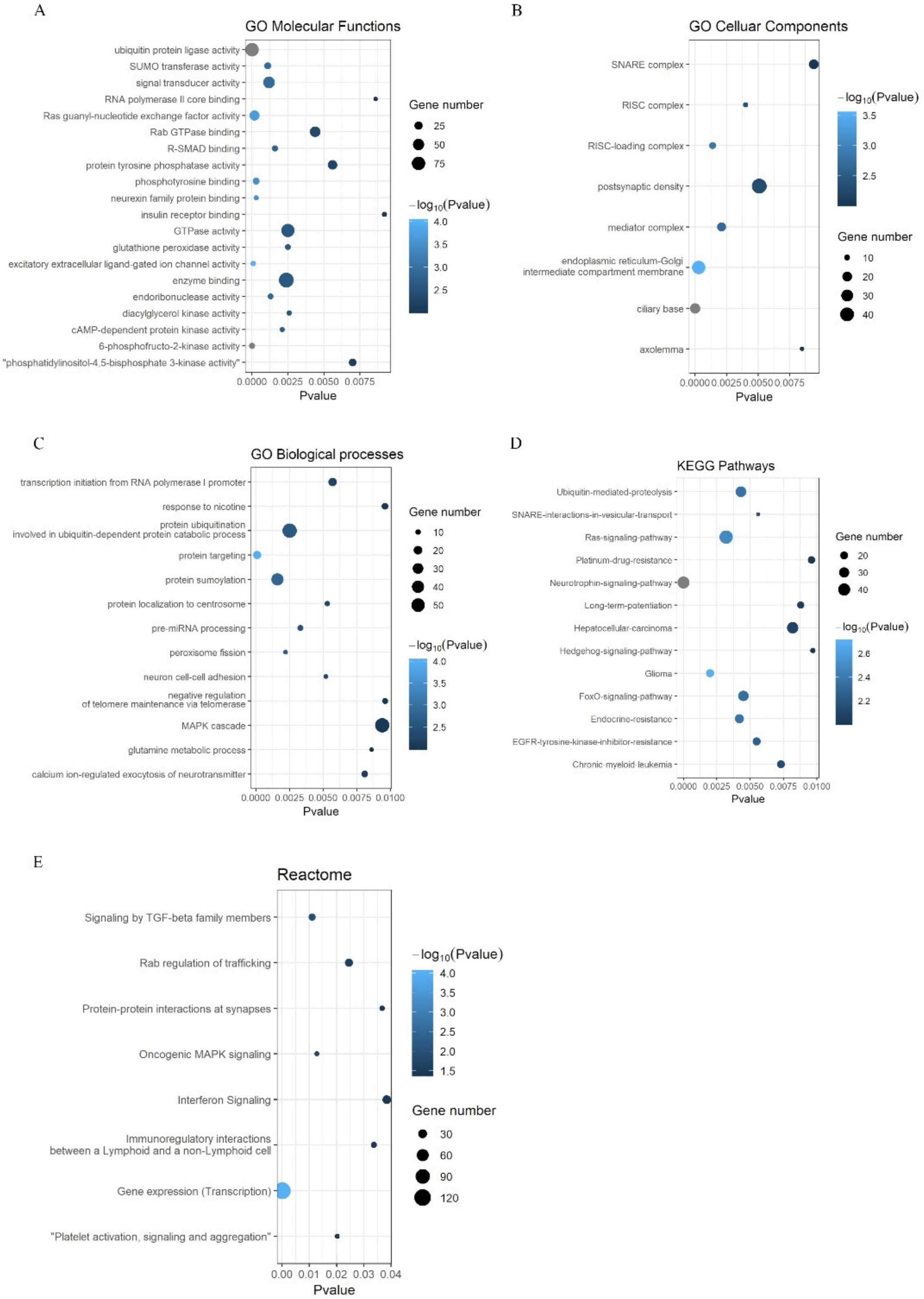

Figure S3. Gene ontology and KEGG pathway enrichment analysis of predicted targets of miR-654-5p by miRWalks3.0. the predicted genes (score>0.95) of miR-654-5p by new version 3.0 of miRWalks were all chosen to perform GO annotation and KEGG pathway enrichment analysis

results to . Each bubble represents an enriched term, its size represents the counts of involved genes. Lighter colors indicate smaller P values. (A) Enriched terms of GO molecular functions. (B) Enriched terms of GO cellular compounds. (C) Enriched terms of GO biological processes. (D) Enriched terms of KEGG pathway. (E) Enriched terms of Reactome.

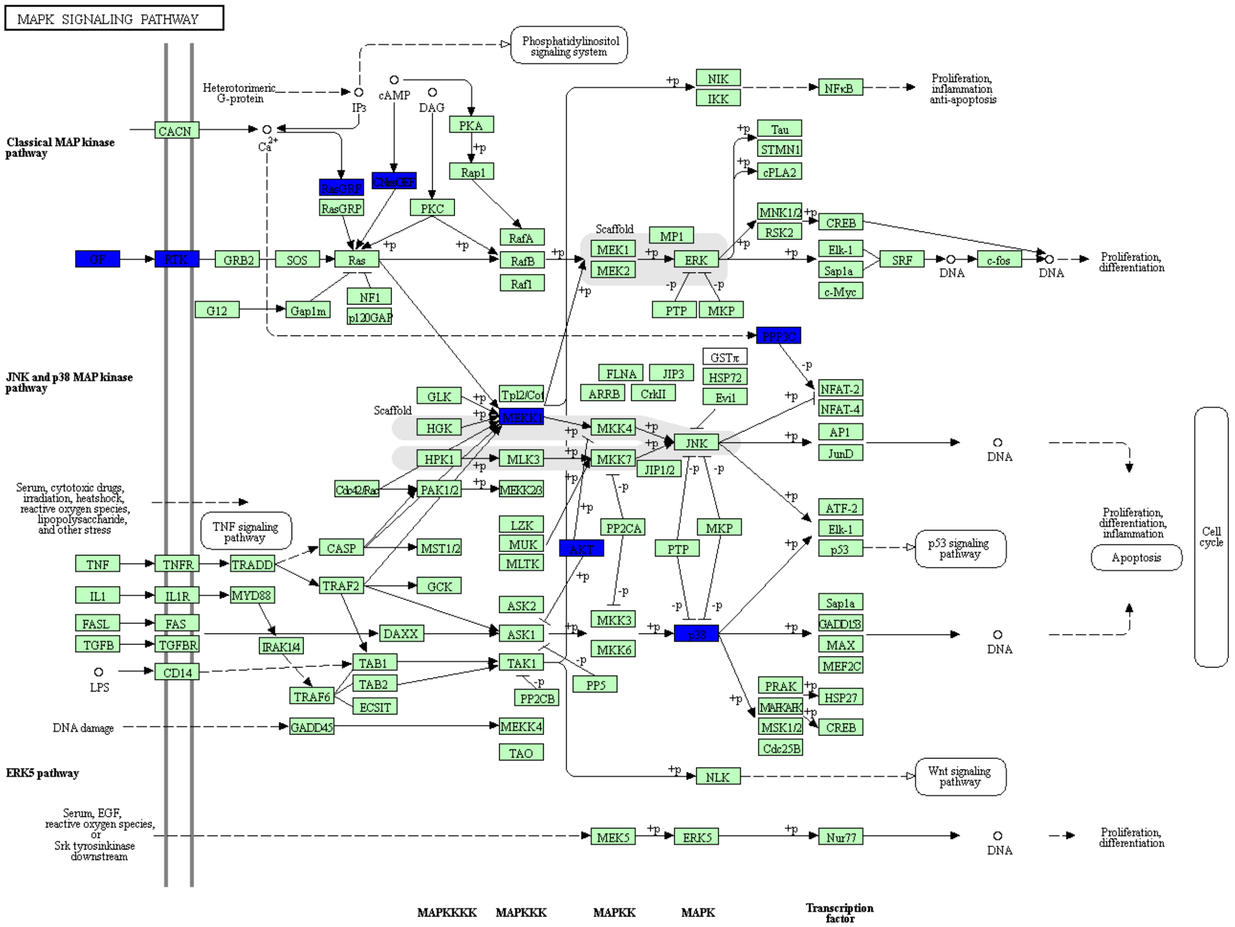

Figure S4. The inhibitory influence of miR-654-5p on MAKP pathway in *Homo sapiens*. the 275 predicted genes of miR-654-5p were mapped to KEGG pathway. Genes regulated by miR-654-5p were colored in blue.

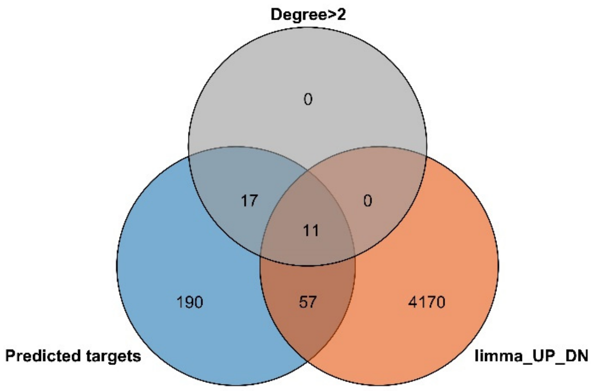

Figure S5. The hub genes of miR-654-5p in LUAD. 275 predicted genes were screened by the criterion of high connection in PPI network (Degree>2) and differential expressing (by R package limma with corrected p-value<0.05) in TCGA LUAD database[3].

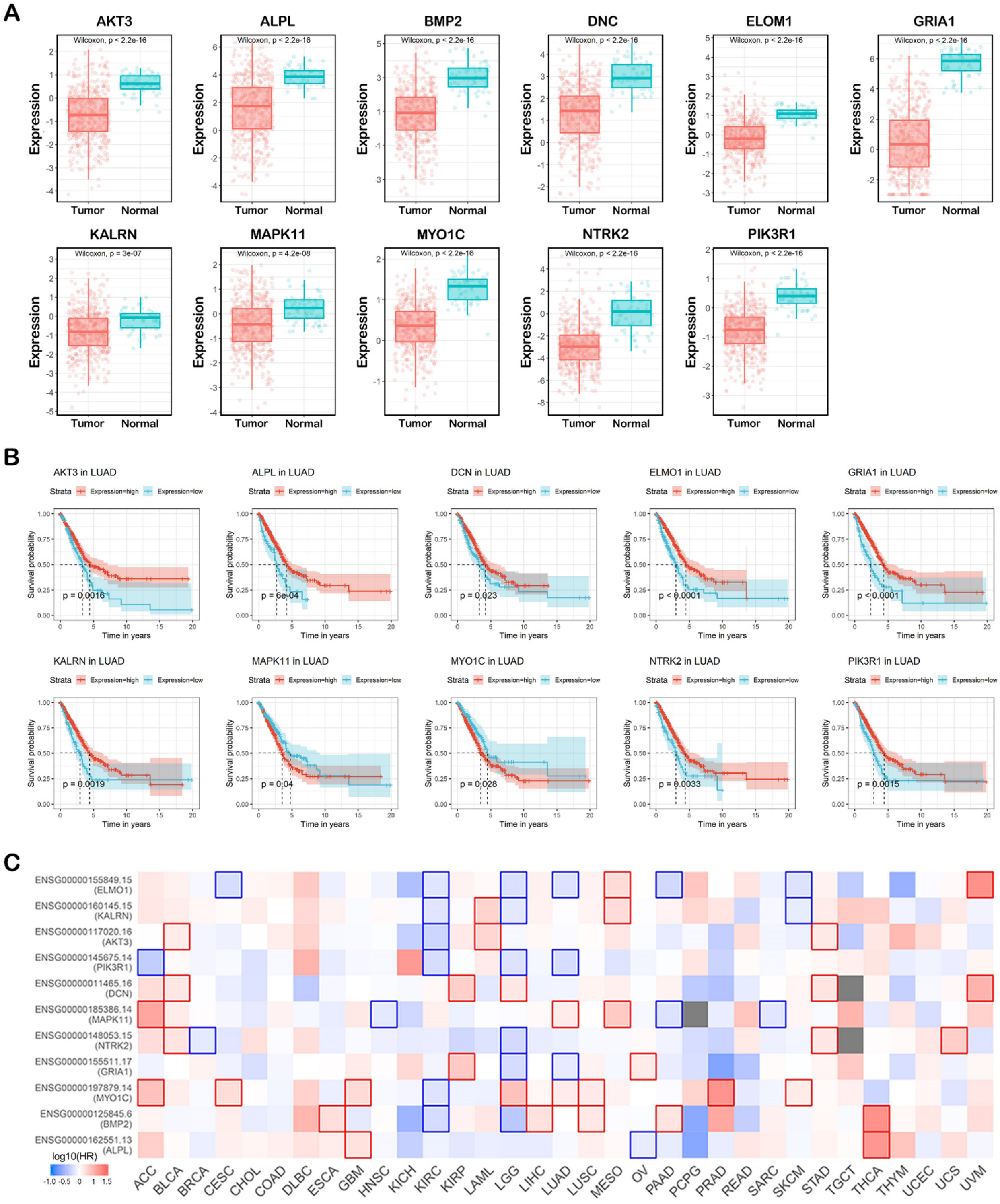

Figure S6. The expression and prognostic analysis of 11 hub targets in cancers (A) The differential expression analyses of hub genes in LUAD were based on pan-cancer batch effect normalized TCGA samples, Wilcoxon test was used to compare the mean between the cancer group and the normal tissue group. (B) The OS rate correlated with 11 hub targets by Kaplan-Meier survival analysis based on the TCGA LUAD database. A log rank p <0.05 was considered to indicate a statistically significant difference. Those with p>0.05 are not shown. (C) The heatmap of the pan-cancer OS rate of 11 hub targets by Kaplan-Meier survival

analysis based on TCGA samples by GEPIA. A log rank $p < 0.05$ was considered to indicate a statistically significant difference and are framed in red (positively correlated) or blue (negatively correlated).

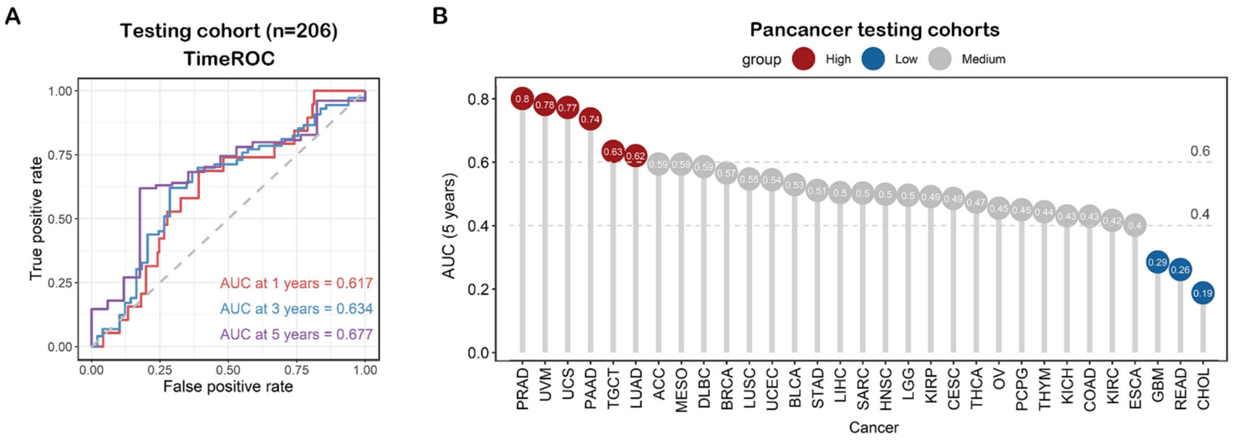

Figure S7. The performance of the risk score model. (A) ROC curve showing the model performance in testing cohort. (B) The risk score model was applied to Pan-cancer mRNA dataset.

Table S1. The abbreviation of TCGA names

Abbr	Full name	Samples(n=)
BLCA	Bladder Urothelial Carcinoma	436
BRCA	Breast invasive carcinoma	1236
CESC	Cervical squamous cell carcinoma and endocervical adenocarcinoma	312
CHOL	Cholangiocarcinoma	45
COAD	Colon adenocarcinoma	545
DLBC	Lymphoid Neoplasm Diffuse Large B-cell Lymphoma	48
ESCA	Esophageal carcinoma	204
GBM	Glioblastoma multiforme	602
HNSC	Head and Neck squamous cell carcinoma	604
KICH	Kidney Chromophobe	91
KIRC	Kidney renal clear cell carcinoma	944
KIRP	Kidney renal papillary cell carcinoma	352
LAML	Acute Myeloid Leukemia	200
LGG	Brain Lower Grade Glioma	529
LIHC	Liver hepatocellular carcinoma	438
LUAD	Lung adenocarcinoma	641
LUSC	Lung squamous cell carcinoma	623
MESO	Mesothelioma	87
OV	Ovarian serous cystadenocarcinoma	604
PAAD	Pancreatic adenocarcinoma	196
PCPG	Pheochromocytoma and Paraganglioma	187
PRAD	Prostate adenocarcinoma	566
READ	Rectum adenocarcinoma	183
SARC	Sarcoma	271
SKCM	Skin Cutaneous Melanoma	479
STAD	Stomach adenocarcinoma	511
TGCT	Testicular Germ Cell Tumors	139
THCA	Thyroid carcinoma	580
THYM	Thymoma	126
UCEC	Uterine Corpus Endometrial Carcinoma	583
UCS	Uterine Carcinosarcoma	57
UVM	Uveal Melanoma	80

Table S2. The identification of key targets of miR-654-5p in various cancers.

ACC	BLCA	BRCA	CESC	COAD	DLBC	ESCA
ASF1B	ATP8B2	-	PEAR1	-	POLR2F	ATF3<br>GNL3L<br>ANGPT2
**GBM**	**HNSC**	**KICH**	**KIRC**	**KIRP**	**LAML**	**LGG**
MYO1C<br>TMEM150A	-	-	-	NRN1<br>MFAP4	RNF8<br>MRPL49<br>ASF1B	MAP3K1<br>PBX3
**LIHC**	**LUAD**	**LUSC**	**OV**	**PAAD**	**PRAD**	**READ**
-	PPT2<br>PIK3R1	CADM3<br>BMP2<br>FLT4<br>ASF1B	ALPL<br>GALNT10<br>PEAR1	ATP8B2<br>AP3S1<br>ASF1B	-	ZNF493<br>NICN1
**SKCM**	**STAD**	**TGCT**	**THCA**	**THYM**	**UCEC**	**UCS**
-	EFNA3<br>HIF3A<br>AFF3<br>UBE2QL1<br>ASF1B	PEAR1<br>ZNF385B	-	SLC7A6<br>HDAC7	PEAR1	SYT13

Reference

1. Ai C, Kong L. CGPS: A machine learning-based approach integrating multiple gene set analysis tools for better prioritization of biologically relevant pathways. J Genet Genomics 2018;45:489-504.

2. Xie C, Mao X, Huang J, Ding Y, Wu J, Dong S, Kong L, Gao G, Li CY, Wei L. KOBAS 2.0: a web server for annotation and identification of enriched pathways and diseases. Nucleic Acids Res 2011;39:W316-22.

3. Tang Z, Kang B, Li C, Chen T, Zhang Z. GEPIA2: an enhanced web server for large-scale expression profiling and interactive analysis. Nucleic Acids Res 2019.
